# An epithelial-mesenchymal plasticity signature identifies two novel LncRNAs with the opposite regulation

**DOI:** 10.3389/fcell.2022.885785

**Published:** 2022-09-01

**Authors:** Pranjal Kumar, Seema Khadirnaikar, Nikita Bhandari, Annesha Chatterjee, Sudhanshu Shukla

**Affiliations:** ^1^ Department of Biosciences and Bioengineering, Indian Institute of Technology Dharwad, Dharwad, Karnataka, India; ^2^ Department of Electrical Engineering, Indian Institute of Technology Dharwad, Dharwad, Karnataka, India

**Keywords:** EMT, lncRNAs, PI3K pathway, cellular plasticity, cancer classification

## Abstract

The epithelial to mesenchymal transition (EMT) is crucial for cancer progression and chemoresistance. EMT is a dynamic process with multiple phases that change cell migration and invasion activity. We used pan-cancer expression data to find 14-LncRNAs that had a high correlation with the EMT markers VIM, CDH1, FN1, SNAI1, and SNAI2. The expression of 14 EMT-associated LncRNA, which also showed high cancer specificity, was used to calculate the pan-cancer EMT score. The EMT score was then applied to the 32 cancer types to classify them as epithelial, epithelial-mesenchymal, mesenchymal-epithelial, or mesenchymal tumors. We discovered that the EMT score is a poor prognostic predictor and that as tumor mesenchymal nature increased, patient survival decreased. We also showed that the cell of origin did not influence the EMT nature of tumors. Pathway analysis employing protein expression data revealed that the PI3K pathway is the most crucial in determining the EMTness of tumors. Further, we divided CCLE-cell lines into EMT classes and discovered that mesenchymal cells, which exhibited higher PI3K pathway activation, were more sensitive to PI3K inhibitors than epithelial cells. We identified Linc01615 as a mesenchymal LncRNA whose expression significantly correlated with survival in several cancer types. We showed that Linc01615 is regulated by the TGFβ-STAT3 pathway in a feedback loop. Knockdown of Linc01615 inhibited cell proliferation and migration by regulating the PI3K pathway and mesenchymal markers. We also identified RP4-568C11.4 as an epithelial cancer marker. We showed that knocking down RP4-568C11.4 decreased cell growth but not migration. In addition, we discovered that ESR1 regulates RP4-5681C11.4 in breast cancer. Taken together, we have developed a pan-cancer EMT signature. Also, we found two new LncRNAs that have different effects on cancer development and EMT.

## Introduction

Cancer is the second most common cause of death globally (Rana et al., 2021). According to a recent study, 19.3 million people were diagnosed, and 10 million died in 2020 due to cancer (Sung et al., 2021). Disease progression, metastasis, and chemoresistance are the primary cause of the poor outcome of the majority of cancer patients (Zaorsky et al., 2017). Metastasis, which includes cell migration and invasion, plays an essential role in cancer progression (Novikov et al., 2021). For the movement, cells may need to acquire a mesenchymal phenotype through epithelial-mesenchymal transition (EMT), which is also associated with chemoresistance and cell proliferation (Dudas et al., 2020). Studies on mouse models have shown that EMT inhibition makes cells more resistant to chemotherapeutic drugs (Fischer et al., 2015; Zheng et al., 2015). During EMT, epithelial cells gradually acquire a mesenchymal phenotype, including enhanced migration capabilities, apoptosis resistance, etc. (Kalluri and Weinberg, 2009). However, EMT shows a range of phenotypes, possibly due to variation in the cells’ microenvironment and genetic and epigenetic composition (Taki et al., 2021). Also, as EMT is a dynamic and reversible process, many cancers acquire some mesenchymal phenotype and maintain some epithelial nature (Williams et al., 2019). Hence, we expect to see tumors with complete mesenchymal, complete epithelial, or mixed phenotypes; for example, Oesophageal squamous carcinoma has four different EMT phenotype types (Sung et al., 2011). Since EMT is associated with proliferation, chemoresistance, and metastasis, it is crucial to identify each tumor’s EMT properties (here on refers to EMTness). This classification may also help in the identification of novel treatment options.

Generally, the presence of mesenchymal markers Vimentin (VIM), fibronectin (FN1), Snail (SNAI1), Slug (SNAI2), and absence of E-Cadherin (CDH1) expression defines the mesenchymal phenotype, and expression of these markers in vice versa fashion defines the epithelial phenotype. However, recent guidelines have suggested that EMT status should not be defined based on a small number of molecular markers (Yang et al., 2020). Also, most proteins are not cancer and tissue-specific and show variable expression (Iyer et al., 2015). In comparison, Long non-coding RNAs (LncRNA) are known to have striking tissue and cancer-specific expression (Iyer et al., 2015). LncRNAs are >200 nt long RNA molecules that lack the capacity to code. Recent research has linked LncRNAs to a variety of cellular processes through a range of mechanisms. HOTAIR, a well-studied LncRNA, for example, influences cancer growth and metastasis in a variety of cancer types (Hajjari and Salavaty, 2015). GAS5, MEG3, NBAT1, PTENP1, ANRIL, MALAT, NKILA, and many other LncRNAs have been extensively studied and are known to promote cancer characteristics such as DNA damage, immunological scape, metabolic dysfunction, stemness, and so on (Pardini et al., 2019). One of the most critical pathways in EMT control is the TGFβ pathway. Many LncRNAs are known to modulate EMT through the TGFβ pathway. PVT1, which is overexpressed in many cancer types, promotes EMT through altering SMAD2/3 phosphorylation and activation (Zhang et al., 2018). SNGH1 is another LncRNA that regulates the TGFβ pathway. SNGH1 acts as a sponge for many miRNAs that modulate the TGFβ pathway (Wang et al., 2018). The LncRNAs stimulated by TGFβ, ATB, and HIT function as a sponge, suppressing the action of the EMT transcription factors ZEB1 and ZEB2 (Yuan et al., 2014; Jia et al., 2016). MEG3 regulates the TGFβ *via* an RNA-DNA triplex structure (Mondal et al., 2015). Similarly, PNUTS has been linked to the control of breast cancer metastasis *via* the regulation of the EMT process (Grelet et al., 2017). In addition, HOTAIR has been demonstrated to influence the EMT process in a range of cancers (Gupta et al., 2010; Padua Alves et al., 2013). Many additional LncRNAs have been linked to the overall EMT process. However, since EMT is a multi-layered process, the involvement of LncRNA in EMT regulation deserves more investigation. As a result, in this study, we used PAN cancer expression data to identify EMT-associated LncRNAs and construct the EMT score. The EMT score was used to categorise TCGA tumor and CCLE cell lines into several EMT classes. We also discovered two new LncRNAs, Linc01615 and RP4-568C11.4, that inhibit cell growth and migration in opposing ways. The workflow used in this study is given in Supplementary Figure S1.

## Materials and methods

### Data sets and epithelial to mesenchymal transition score analysis

**FIGURE 1:**
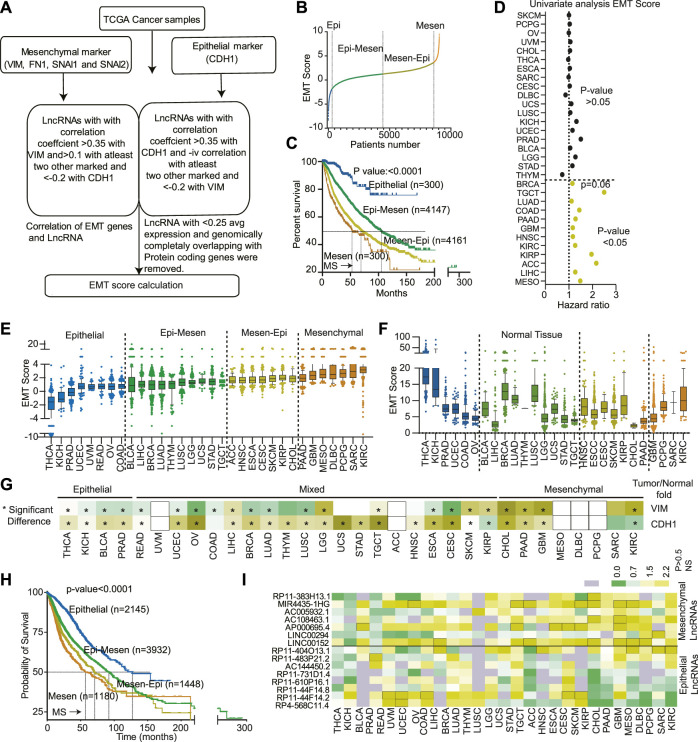
Classification of 32 cancer types based on EMTness: **(A)** A flow chart showing the steps used to identify the EMT-related LncRNAs. **(B)** An EMT score was developed for each TCGA cancer patient using the formula described in the method section. The plot shows the division of cancer patients into Epithelial (Epi), Epithelial-Mesenchymal (Epi-Mesen), Mesenchymal-Epithelial (Mesen-Epi), and mesenchymal groups. **(C)** The Kaplan- Meier plot showing the differences in survival of the four EMT groups. **(D)** EMT score for each cancer type was used to perform the univariate Cox-regression analysis. The Hazard ratio and *p*-value are depicted. **(E)** The EMT score for 32 TCGA cancer types were plotted in increasing order of average EMT score for each cancer type. The cancer types were then divided into four EMT groups. **(F)** The EMT score for normal samples was calculated and plotted to show an association between cell origin and EMTness of cancer. **(G)** Expression of VIM and CDH1 was compared between normal and tumor of each cancer type and plotted as a heatmap. Yellow is associated with increased expression, and green is associated with decreased expression. * indicates the significant comparisons. **(H)** The survival of cancers based on EMTness, as identified in Figure 2E, compared using the Kaplan-Meier plot. **(I)** The fold change in expression between normal and cancer of each of the 14 LncRNAs was calculated and plotted. Green indicates significant decreases, and yellow indicates a significant increase in expression in the tumor compared to normal. The grey indicates non-significant differences in expression.

Expression analysis was done using the RNA-sequencing data from TCGA-GDC. RNA-seq data represented by FPKM values for all the samples from the TCGA project were downloaded from the GDC website using the “TCGAbiolinks” library in R for each tumor type separately. Tumor and normal samples from each tumor type were segregated based on the barcode and used for further analysis. Survival data for all the tumor types were also downloaded from the firehose repository. The identification of EMT LncRNA and calculation of EMT score is described in Figure 1A. The expression of VIM, FN2, SNAI1, SNAI2, and CDH1 was correlated with all the annotated LncRNA in TCGA cancers. The LncRNAs having a correlation coefficient of more than 0.35 with VIM and a correlation coefficient of >0.1 with at least two of FN1, SNAI1, SNAI2 and a correlation coefficient of < −0.2 with CDH1 were considered mesenchymal LncRNA. Oppositely, LncRNAs with correlation coefficient of more than 0.35 with CDH1 and negative correlation coefficient with at least two of FN2, SNAI1, SNAI2 and a correlation coefficient of < −0.2 with VIM were considered epithelial LncRNAs. The overview of this is shown in Figure 1A. For EMT score calculation, the correlation coefficient of each selected LncRNA with CDH1 and VIM1 was added. The values were then multiplied by the expression value of each LncRNA and added together to find the EMT score (Supplementary Table S1). The following formula was used for the EMT score calculation-

$$\begin{array}{l} \text{EMT Score }=\left(\text{RP}11-383\text{H}13\text{.}1\text{ ×}\text{0}\text{.}16\right)+\left(\text{MIR}4435-1\text{HG}\text{ ×0}\text{.}15\right)+\left(\text{AC}005932\text{.}1\times \text{0}\text{.}05\right)+\left(\text{AC}108463\text{.}1\times \text{0}\text{.}15\right)+\left(\text{AP}000695\text{.}4\times \text{0}\text{.}18\right)+\;\left(\text{LINC}00294\times \text{0}\text{.}14\right)\;+\;\\ \left(\text{LINC}00152\times \text{0}\text{.}13\right)+\left(\text{RP}11-404\text{O}13\text{.}1\text{ ×}\text{0}\text{.}09\right)+\left(\text{RP}11-483\text{P}21\text{.}2\times \text{0}\text{.}12\right)+\left(\text{AC}144450\text{.}2\times \text{0}\text{.}04\right)+\;\left(\text{RP11}-731\text{D}1\text{.}4\times \text{0}\text{.}06\right)\;+\left(\text{RP11}-610\text{P}16\text{.}1\times \text{0}\text{.}06\right)\\ +\left(\text{RP}11-44\text{F}14\text{.}8\times \text{0}\text{.}05\right)+\left(\text{RP}11-44\text{F}14\text{.}2\times \text{0}\text{.}05\right)+\left(\text{RP}4-568\text{C}11\text{.}4\times -\text{0}\text{.}048\right) \end{array}$$

We also utilized the microarray and RNA-seq data associated with GSE146066 (Lucciola et al., 2020), GSE151251, and GSE153250 (Broome et al., 2021) datasets.

### Survival, correlation analysis

To understand the association of various factors with prognosis, Cox regression and Kaplan-Meier plot with Mantle-Cox analysis were performed. For univariate Cox regression analysis, EMT score and for multivariate analysis, EMT score, age, gender, and stage were used as co-variates, and a *p*-value <0.05 was considered significant. For Kaplan-Meier, plots patients were divided into various groups based on the EMT score, Cancer types, or expression of novel LncRNAs. The *p*-value<0.05 was considered significant.

For correlation analysis, Graphpad 9.0 software was used. Spearman’s non-parametric method was used to calculate the correlation coefficients.

### Cell culture

The human cancer cell lines MDA-MB-231, 143B, AGS, MCF7, T47D, HCT116, HT29, H23, H460, and H522 cell lines were obtained from the National Centre for Cell Sciences (NCCS), Pune (India). The cells have been authenticated by the NCCS using STR. H1299 cells were purchased from ATCC, United States. Cell were cultured in recommended cell culture media supplemented with fetal bovine serum (10% v/v) and 100 U/mL Penicillin and 100 μg/ml Streptomycin. Cells were grown in a humidified incubator at 37°C in 5% CO_2_/95% atmospheric air. All the cell lines were tested for *mycoplasma* contamination, and experiments were performed in mycoplasma-free cells only.

### Knockdown of Linc01615

The knockdown study was carried out using two different shRNA constructs targeting Linc01615 RNA, along with negative control vector scramble shRNA (Plasmid #1864, and gene). shRNA hairpin sequences were cloned in lentiviral mammalian expression vector pLKO.1 puro (Plasmid #8453, addgene). The sequence pairs for hairpins (target sequences are shown in bold) are: shRNA#1_1 5′-CCG​GAC​GTC​TGC​TAC​GAA​CTC​AAT​TCT​CGA​GAA​TTG​AGT​TCG​TAG​CAG​ACG​TTT​TTT​G-3’; shRNA#1_2 5′- AAT​TCA​AAA​AAC​GTC​TGC​TAC​GAA​CTC​AAT​TCT​CGA​GAA​TTG​AGT​TCG​TAG​CAG​ACG​T-3’; shRNA#2_1 5′- CCG​GCA​CTC​CCG​GAG​CAG​CAG​GAA​ACT​CGA​GTT​TCC​TGC​TGC​TCC​GGG​AGT​GTT​TTT​G-3’; shRNA#2_2 5′- AAT​TCA​AAA​ACA​CTC​CCG​GAG​CAG​CAG​GAA​ACT​CGA​GTT​TCC​TGC​TGC​TCC​GGG​AGT​G; Scramble shRNA sequence is 5′-CCT​AAG​GTT​AAG​TCG​CCC​TCG​CTC​GAG​CGA​GGG​CGA​CTT​AAC​CTT​AGG-3’. As per the manufacturer’s recommendation, all the transfections were carried out using Xfect polymer (TaKaRa Bio, cat.#631317). The transfected cells were selected using 2 μg/ml puromycin (Sigma, cat.#P8833) for a minimum of 48 h and used for RNA isolation using TRIzol reagent (Ambion, cat.#15596018), and 250 ng of total RNA was used to synthesize cDNA using iScript RT mix (Biorad, cat.#1708840). 20 ng of cDNA was used to perform the qPCR/semi-qPCR using universal SYBR Green mix (Biorad, cat.#1725271) or Emerald GT PCR mix (TaKaRa, cat.#RR310A).

### PAGE and western blot

A total of 0.3 × 10^6^ to 0.6 × 10^6^ cells were harvested for protein isolation. Briefly, they were trypsinized and collected in separate micro-centrifuge tubes. They were washed with cold PBS and lysed in a lysis buffer (Pierce IP Lysis Buffer: 87,787) supplemented with a 1X protease inhibitor cocktail (Halt protease inhibitor cocktail: 87,786). The cell lysates were centrifuged at 13,000xg for 15 min, and supernatants were collected. The total protein concentration was estimated before loading onto polyacrylamide gel by the BCA protein assay kit (Pierce: 23,227) and a total of 30 µg of cell lysates from different experimental cells were loaded and resolved in 12% polyacrylamide gel [Biorad TGX FastCast Acrylamide Kit 12%: 161-0175)]. The gel was blotted in the PVDF membrane (Biorad: 1620177), and the membrane was blocked using a 5% blocker solution (Blotting Grade Blocker, Biorad: 1706404). The membrane was cut as per molecular size of proteins and incubated with PARP1 (proteintech: 66520-1-Ig), VIM (Invitrogen: MA5-16409), SNAI1 (proteintech: 13099-1-AP), SNAI2 (proteintech: 12129-1-AP), AKT1 (ABclonal: A10605), phosphor-AKT-S473 (ABclonal: AP1208) and internal control Actin B (ABclonal: AC004) or GAPDH (ABclonal: AC002) antibodies. The membrane was later incubated with HRP-tagged goat anti-mouse (Biorad: 172-1011) or anti-rabbit (Invitrogen: 31,460) secondary antibodies. All the steps were followed by washing with TBS (Biorad: 1706435) consisting of 0.1% Tween 20 (Polysorbate 20, MP Biomedicals: 103,168). The bound antibody complexes were detected with ECL Western Blotting substrate (Pierce: 32,209) or femtoLUCENT PLUS HRP chemiluminescent reagents (G Biosciences: 786-003).

### PCR/qPCR

The polymerase chain reactions were carried out using the Emerald GT PCR mix (TaKaRa Bio: RR310A) in a thermal cycler (Biorad). The PCR samples were checked for their amplicon size and utilized for semi-qPCR using agarose gel electrophoresis. For real-time analysis, universal SYBR Green mix (Biorad: 1725271) was used, and PCR was carried out in Biorad CFX96 using two-step amplification and melt curve protocol. The qPCR analysis was performed following the delta-delta Ct value and plotted.

### Proliferation assay

The cell proliferation assay was carried out using MTT [3-(4,5-dimethylthiazol-2-yl)-2,5-diphenyl-tetrazolium bromide] (Sigma, cat.#). Briefly, all the experimental cells were seeded in a 96-well plate at a density of 2,000 cells/well in triplicate for 4 days. MTT was added to the well every 24-h and incubated at 37°C and 5% CO_2_ (95% atmospheric air) in a humidified incubator for 4 h. The blue-colored formazan complex was dissolved in DMSO (cat.#) and recorded the absorbance at 570 nm.

### Wound healing assay

Cells were seeded in a 24-well plate at a density of 0.3 × 10^6^ cells/well and allowed them to adhere and grow up to ≥95% confluency. Later, the wound was generated using a 200 µl sterile microtip, and the wound size was measured every 12-h up to 36-h. The wound closure was normalized and plotted.

### Colony suppression assay

Cells were seeded in a 12-well plate at a density of 300 cells/well in duplicate and allowed then to grow for about 2 weeks. Later, the media was removed and washed with PBS and stained in 0.25% crystal violet (cat.#) solution. The image was captured.

### TGF-β and STAT-3 inhibitor treatment

MDA-MB-231 and 143B cells were treated with TGF-β1 inhibitor (Selleckchem: S2805) and STAT-3 inhibitor Niclosamide (Selleckchem: S3030). The TGF- β1 inhibitor was used at 10 µM concentration, whereas STAT3 inhibitor was used at 10 µM for 24 h. Also, recombinant TGF- β1 (R&D Systems: 7754-BH-005/CF) was used at 2.5 ng/ml for 24 h. The recombinant TGF- β1 and STAT3 inhibitors were also used in combination at the same concentration for 24 h. Later, cells were harvested and utilized for qRT-PCR.

### Drug sensitivity assay

To study the effect of various drugs/small molecules on cell lines featuring epithelial and/or mesenchymal characteristics (Epithelial: AGS and MCF7; Mesenchymal: 143B), different concentrations of each drug was formulated and used for treatment. Briefly, ∼5,000 cells were plated in 96-well plate and grown for ∼24 h. Later, the cells were treated with different concentrations of each drug (in duplicate) and after 48 h their viability was assayed using MTT (as described previously). The absorbance readings were normalized and plotted.

## Results

### Identification of epithelial to mesenchymal transition associated LncRNAs

The epithelial-mesenchymal transition (EMT) is critical in cancer progression and metastasis (Yang et al., 2020; Novikov et al., 2021). We hypothesized that LncRNAs with a high association to EMT marker expression and tumor specificity could aid in the categorization of epithelial and mesenchymal phenotypes. Hence, we analyzed TCGA expression data from 32 cancer types (∼9,000 patient samples) to identify LncRNAs associated with EMT markers, VIM, FN1, SNAI1, SNAI2, and CDH1 (Figure 1A, and material and method). We identified mesenchymal and epithelial LncRNA (EMT LncRNA) using the method described in the material and method section (Supplementary Figure S2A, and material and method). To further understand the overall function of these EMT LncRNAs, we performed guilt by association analysis on protein-coding genes (PcGs) with high correlation (identified using the GePIA2 database) with EMT LncRNAs. The analysis showed that mesenchymal LncRNAs were associated with cancer progression pathways (Supplementary Figure S2B). Epithelial LncRNAs, on the other hand, have been connected to epithelial growth, transport, and metabolic signalling (Supplementary Figure S2B). Interestingly, a pan-cancer analysis, tumor vs. normal comparison revealed that mesenchymal LncRNAs are frequently overexpressed in tumors and linked to poor survival (Supplementary Figures S2C,D). On the other hand, epithelial LncRNAs were downregulated or marginally upregulated and associated with better prognosis (Supplementary Figures S2C,D).

### Classification of tumors in epithelial to mesenchymal transition classes

Next, we sought to classify tumor based on their EMT potential based on the expression of EMT LncRNA identified in previous section. An EMT score was calculated by adding the weighted expression data of the LncRNAs as described in the material and method section and illustrated in Figure 1A. EMT score was then used to classify the tumors into Epithelial, Epithelial-Mesenchymal, Mesenchymal-Epithelial, and mesenchymal groups (Figure 1B and Supplementary Table S1). The Kaplan-Meier analysis revealed that patients in the epithelial group survived much better (MS-unidentified) than those in the mesenchymal group, which had a very dismal survival rate (MS-52.8 months) (Figure 1C). The other two groups had intermediate survival, with epithelial-mesenchymal patients outliving mesenchymal-epithelial patients (Median survival 106.66 vs. 70.66 months) (Figure 1C). Next, to understand the association of EMT score with individual cancer types, we performed a univariate Cox regression analysis of EMT scores. The results identified the EMT score as significant predictor of poor survival in the 12 cancer types (Figure 1D). Furthermore, to find the prognostic independence of the EMT score in these 12 cancer types, a multivariate analysis with age, stage, gender, and EMT score was performed. The analyses revealed that except for KIRP (*p*-value 0.2), GBM (*p*-value 0.08), LUAD (*p*-value 0.1) and TGCT (*p*-value 0.09), in other eight cancers, EMT score was independent prognosticator of survival (*p*-value<0.05), (Supplementary Table S3).

### Classification of cancer types in epithelial to mesenchymal transition classes

In the previous section, we identified EMT classes across the cancer type. Next, we aim to classify different cancer types based on EMT scores. We calculated the average EMT score for each cancer type and ranked them based on it. Interestingly, we discovered variability in EMTness between cancer types and also among tumors of the same type (Figure 1E). Certain cancers, such as THCA, KICH, BLCA, and PRAD, exhibited a significantly high epithelial phenotype (Figure 1E). In contrast, malignancies such as PAAD, GBM, MESO, DLBC, PCPG, SARC, and KIRC were mostly mesenchymal in origin (Figure 1E). Other cancer types showed a mixture of epithelial and mesenchymal characteristics, classified as epithelial-mesenchymal and mesenchymal-epithelial tumors (Figure 1E). We also computed the EMT score for the normal tissues corresponding to each cancer type. We found no association between the EMTness of normal and equivalent malignancy, suggesting that the EMT nature of the tumor is independent of the tissue of origin (Figure 1F). Interestingly, compared to corresponding normal, epithelial cancer types showed higher expression of CDH1 and lower expression of VIM. Similarly, mesenchymal cancers showed higher expression of VIM in comparison to their respective normal (Figure 1G). CDH1 was, however, consistently overexpressed in most of the cancer types (Figure 1G). Additionally, tumors with a high epithelial component had a considerably longer median survival (undefined) compared to tumors with a strong mesenchymal component (median survival of 64 months) (Figure 1H). As expected, epithelial-mesenchymal and mesenchymal-epithelial tumors showed intermediate survival rates, with epithelial-mesenchymal patients living significantly longer (Median survival 92.1 vs. 70.66 months) (Figure 1H). Also, differential expression analysis showed overexpression of mesenchymal LncRNAs in mesenchymal tumors, and epithelial LncRNA showed higher expression in epithelial tumors (Figure 1I).

### PI3K pathway plays a crucial role in mesenchymal phenotype determination

**FIGURE 2:**
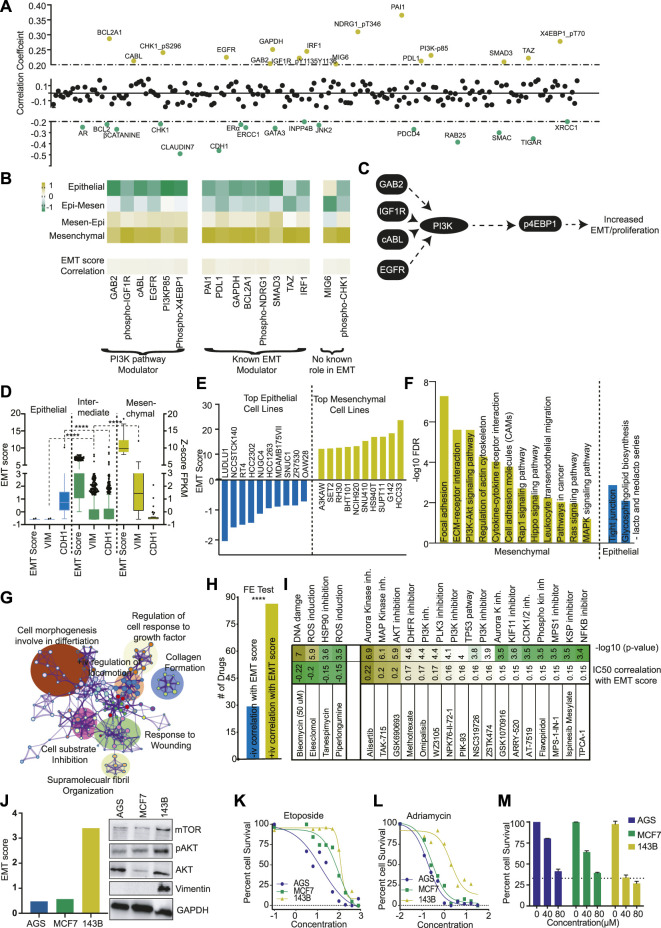
Pi3K pathway plays a major role in mesenchymal phenotype. **(A)** The correlation of EMT score with protein expression data of TCGA patients was calculated and plotted. The Yellow dots represent the positive correlation (>+0.2), and the green dots represent the negative correlation (<−0.2). **(B)** The average expression of proteins with a positive correlation with the EMT score was plotted as a heatmap for each EMT class. The proteins are divided into three classes depending upon their role in the PI3K pathway and EMT. **(C)** A diagram showing the PI3K pathway modulator with a positive correlation with the EMT score. **(D)** EMT score for each CCLE cell line was calculated, and cells were divided into epithelial, mixed, and mesenchymal groups. The expression of VIM and CDH1 was compared in three groups and plotted. **(E)** EMT score for each CCLE cell line was calculated, and the EMT score of the top 10 epithelial and mesenchymal cells was plotted. **(F)** The GSEA analysis was done to show the activation of various pathways in mesenchymal and epithelial cell lines. **(G)** The differential expression analysis was done between mesenchymal and epithelial cells, and differentially expressed genes were used from the pathway analysis using Metascape. The pathway network was plotted as identified in the analysis. **(H)** The EMT score was correlated with the IC50 value of drugs as obtained from Genomic drug sensitivity in cancer (GDSC) databases. The drugs were divided into two groups (positive and negative correlation with EMT score). Fisher’s exact test suggests that significantly more drugs showed increased IC50 in high EMT cells. **(I)** The correlation coefficient of drugs with high correlation (±0.15) with EMT scores were identified and plotted. **(J)** Three cell lines AGS, MCF (epithelial), and one 143B (mesenchymal) were selected, and levels of mTOR, pAKT, AKT, and VIM were determined with western blot. **(K, L)** The effect of DNA damaging compounds **(I)** Etoposide **(J)** Adriamycin was determined in AGS, MCF7 (epithelial), and 143B (mesenchymal cells) and plotted to show the high resistance of 143B for DNA damaging compounds. **(M)** The effect of the PI3K inhibitor was measured at two different concentrations to show the high sensitivity of PI3K inhibitors on mesenchymal cell line 143B.

Next, to understand the differences in proteins and protein modifications (changes in phosphorylation levels of proteins) associated with EMT phenotype, we performed a correlation analysis between EMT score and 217 proteins assayed in the TCPA database. The proteins/modifications with ±0.2 correlation coefficient and 0.05 FDR were considered significant. The analysis identified 16 proteins with positive and 16 proteins with negative correlation (Figure 2A). The protein with the highest negative correlation with EMT score was CDH1, validating our initial analysis (Supplementary Figure S3A). Some of the know EMT inhibitors like GATA3, CLDN7, INPP4B, JNK2, etc., were also identified as negatively correlating proteins (Hu et al., 2017; Li et al., 2018; Tang et al., 2019; Bai et al., 2021). Of the 16 positively correlating proteins, 50% were proteins with a well-established role in EMT (Figure 2B). More importantly, six proteins/protein modifications with a strong positive correlation with EMT were identified as modulators of the PI3K pathway (Figures 2B,C). We also compared the protein level data and found that PI3K pathway activators like TSC1, 4EBP1 and p4EBP1, PI3K-p85, RICTOR-pT1135 were significantly increased in high mesenchymal tumors, and PI3K inhibitor like LKB1 and INPP4B expression was significantly decreased (Supplementary Figure S3B) (Hoxhaj and Manning, 2020). These observations suggest that the PI3K pathway plays a crucial role in the mesenchymal phenotype (Figure 2L). The results also indicate that patients with a high EMT phenotype may have high PI3K pathway activation and thus may be more sensitive to PI3K inhibitors.

### High mesenchymal cell lines are highly sensitive to PI3K pathway inhibitors

To understand the role of the PI3K pathway in EMTness, we utilised the cell line models. First, we calculated the EMT score of cell lines from the CCLE database. As expected, the EMT score showed a significant positive correlation with mesenchymal markers and a negative correlation with CDH1 (Supplementary Figure S4A). We classified the cell lines into four classes as TCGA cancer patients samples (Supplementary Table S2). As cell lines are generally selected as models based on epithelial or mesenchymal phenotype, we mixed the two groups in the middle. We further classified the CCLE cells into epithelial, intermediate, and mesenchymal groups (Figure 2D). The cells classified as epithelial cells showed high expression of CDH1 and negligible expression of VIM (Figure 2D). In contrast, mesenchymal cells showed high VIM expression with low expression of CDH1 (Figure 2D). The cells classified as intermediate showed expression of both VIM and CDH1. (Figure 2D). We also listed top cell lines with the highest epithelial and mesenchymal nature for use as EMT models (Figure 2E). Furthermore, GO analysis showed that mesenchymal cells expressed genes associated with Adhesion, ECM-receptor interaction, PI3K signalling, MAPK signalling, etc.(Figure 2F). Similarly, Metascape network analysis showed that the genes differentially expressed between mesenchymal and epithelial cell lines were enriched in cell movement and wound healing processes (Figure 2G). Next, we analysed drug sensitivity data from the DGSC database and found that, generally, epithelial cells were more sensitive to drugs than mesenchymal cells (Figure 2H). However, the closer analysis showed that epithelial cells were more sensitive to general chemotherapeutic drugs affecting DNA damage, ROS activation, etc. In contrast, mesenchymal cells were sensitive to pathway inhibitors, specifically the PI3K pathway (Figure 2I). To further validate the findings, we selected 3 cell lines, MCF7, AGS (Epithelial), and 143B (mesenchymal). MCF7 and AGS showed low EMT scores and low expression of VIM and PI3K pathway markers compared to 143B cells (Figure 2J). As found in DGSC data, MCF7 and AGS cells were more sensitive to DNA damaging agents, Etoposide, and Adriamycin than 143B cells (Figures 2K,L). In contrast, 143B cells were significantly more sensitive to PI3K inhibitor LY294002 at tested concentrations (Figure 2M). These results support the findings from cancer patients’ data described in the previous section, which suggest that tumors with high mesenchymal nature (higher EMT score) may be more sensitive to PI3K inhibitors due to higher activation of the PI3K pathway.

### Long non-coding RNA Linc01615 is strongly associated with mesenchymal phenotype-

**FIGURE 3:**
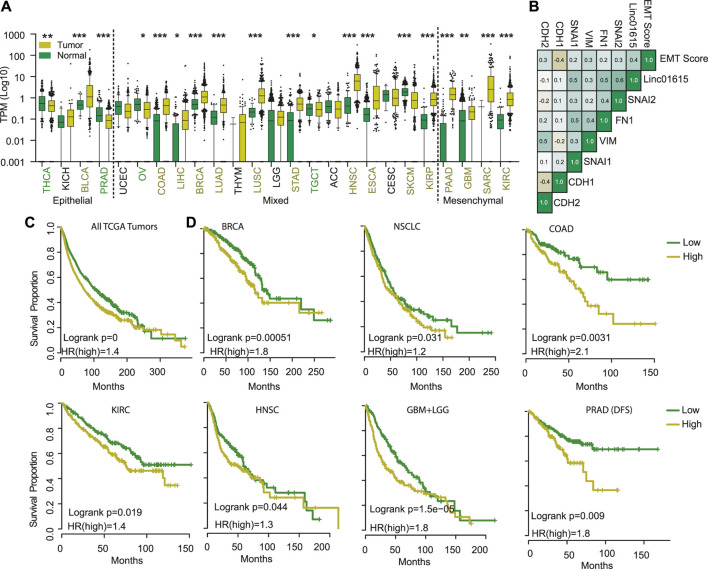
Linc01615 is a mesenchymal LncRNA. **(A)** The correlation of Linc01615 expression was done with EMT score and EMT markers and shown. **(B)** Expression of Linc01615 was compared in all the TCGA cancer types and plotted. **(C)** All the TCGA patients were combined and divided into patients with high and low Linc01615 expression, and a Kaplan-Meier analysis was performed. **(D)** Individual cancer types were divided into low and high Linc01615 groups, and survival was compared using Kaplan-Meier analysis.

Next, we compared the gene expression of top mesenchymal and top epithelial cells to identify genes associated with epithelial and mesenchymal cell lines (Supplementary Figure S4B). The genes overexpressed in mesenchymal cells showed significant enrichment of GO terms associated with ECM organization, wound healing, etc. (Supplementary Figure S4C). In comparison, genes overexpressed in epithelial cells showed significant enrichment of GO terms associated with epithelial cell development and differentiation (Supplementary Figure S4C). Further, we checked the expression of top differentially expressed LncRNAs between mesenchymal and epithelial cells (Supplementary Figure S4D). The analysis identified 16 LncRNAs with high specificity to mesenchymal (10 LncRNA) or epithelial (6 LncRNAs) cells. We performed survival analysis across TCGA cancer and found that two LncRNAs, Linc01615 (RP11-417E7.1) and AP000695.6, were associated with poor prognosis in more than 15% of the tumor types (Supplementary Figure S4E). Both of the LncRNA were overexpressed and prognostic in multiple cancer types (Figure 3A and Supplementary Figure S5A). However, Linc01615 showed consistently strong expression patterns and mesenchymal association; hence, we studied it in detail. Correlation analysis of Linc01615 with EMT markers showed a significant positive association between Linc01615 and EMT score, SNAI1, SNAI2, VIM, and FN1 (Figure 3B). Also, the expression of Linc01615 was high in mesenchymal tumors (PAAD, GBM, SARC, and KIRC) and lowered in epithelial tumors (THCA, KICH, PRAD, and except BLCA) (Figure 3A). Even in mixed-class cancers, Linc01615 showed significantly higher expression than corresponding normal (Figure 3A). The Kaplan-Meier analysis validated the prognostic association of Linc01615 expression with poor survival in multiple cancer types (Figures 3C,D).

### Linc01615 is regulated *via* TGFβ signalling

**FIGURE 4:**
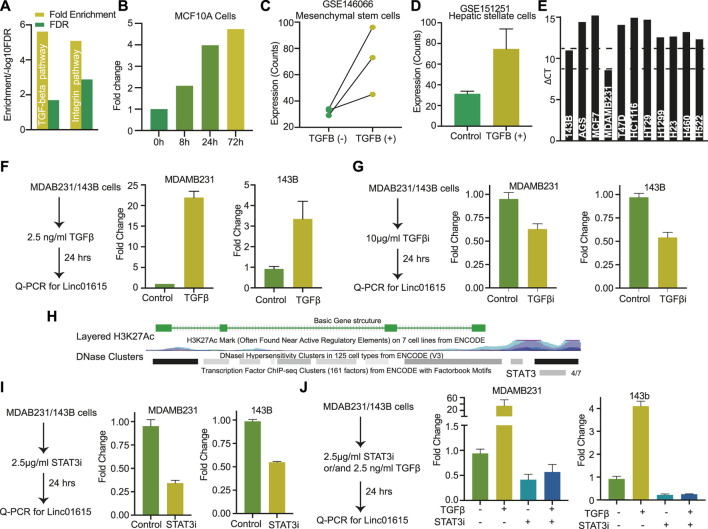
Linc01615 is regulated by the TGFβ pathway: **(A)** The genes with a high correlation coefficient with Linc01615 expression were used for KEGG pathway analysis. The Fold enrichment and FDR of enriched pathways are depicted. **(B)** Expression level of Linc001615 in MCF710A cells after treatment with TGFβ at mentioned time points. Expression of Linc01615 after TGFβ treatment in **(C)** mesenchymal stem cells and **(D)** hepatic Stellate cells. **(E)** Expression of Linc01615 in different cells as identified using semi-quantitative PCR. **(F)** Expression of Linc01615 RNA after TGFβ treatment in MDAMB231 and 143B cells. **(G)** Expression of Linc01615 RNA after TGFβ inhibitor in MDAMB231 and 143B cells. **(H)** UCSC genome browser image showing the binding site of STAT3 on the promoter of Linc01615. **(I)** Expression of linc01615 RNA after STAT3 inhibitor treatment in MDAMB231 and 143B cells. **(J)** Expression of linc01615 RNA after TGFβ, STAT3 inhibitor, or combined treatment in MDAMB231 and 143B cells.

To understand the mechanism of the Linc01615 function in cancer, we performed the Panther pathway analysis using the genes significantly correlating with Linc01615 expression (correlation coefficient >0.3). The analysis identified significant enrichment of TGFβ and integrin pathways (Figure 4A). Thus, to check the effect of the TGFβ pathway on Linc01615, we downloaded the three RNA-seq based expression data from GEO and analyzed them. The analysis showed that in all the cell types (MCF10A, Mesenchymal Stem cells, and hepatic stellate cells) expression level of Linc01615 is increased after TGFβ treatment (Figures 4B–D). Next, we used MDAMB321 and 143B cells (Figure 4E) and treated them with TGFβ for 24 h (Figure 4F). The quantification of Linc01615 showed a substantial increase in the expression after TGFβ treatment (Figure 4F). Also, treatment of TGFβ inhibitors showed a small decrease in the expression of Linc01615 in both cells (Figure 4G). The canonical pathway of TGFβ regulates EMT *via* SMAD4. Thus, we checked for the binding of the SMAD4 at the Linc01615 promoter using ENCODE data. However, no binding of SMAD4 was seen in any of the cells. However, we noticed non-canonical TGFβ effector STAT3 binding at the promoter of Linc01615 in four out of seven analyzed samples (Figure 4H). Thus, we checked the effect of STAT3 inhibitor on Linc01615 expression in MDAMB231 and 143B cells. Both the cells showed decreased Linc01615 expression after STAT3 inhibitor treatment, confirming the role of STAT3 in Linc01615 expression (Figure 4I). To see the effect of STAT3 inhibitor on TGFβ mediated Linc1615 regulation, we treated the cells with the combination of TGFβ and STAT3 inhibitor (Figure 4J). STAT3 inhibitor decreased the expression of Linc01615 even in the presence of TGFβ, suggesting STAT3 is a downstream regulator of Linc01615 (Figure 4J). These observations confirm that Linc01615 is regulated by the non-canonical TGFβ−STAT3 pathway.

### Linc01615 regulated the growth and migration of cells

**FIGURE 5:**
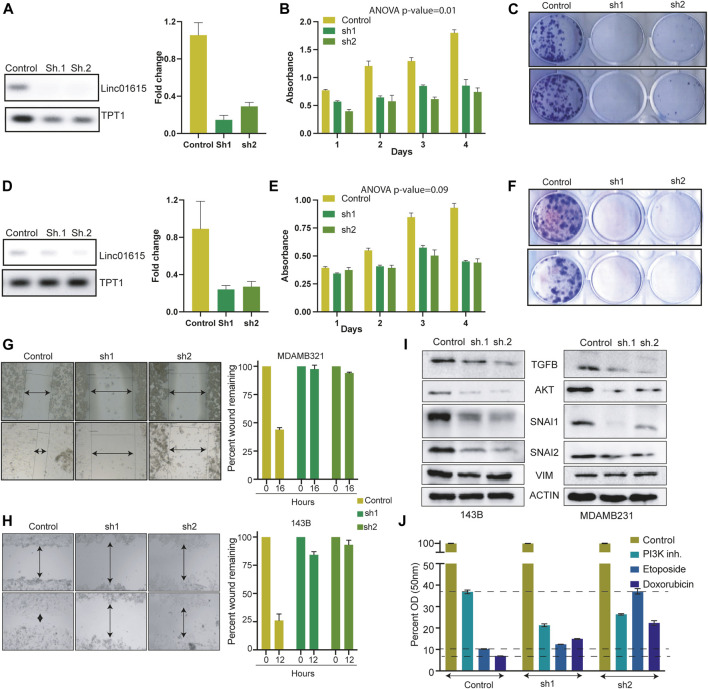
Linc01615 is required for cell proliferation and cell migration: **(A)** Semi-quantitative and Q-PCR data showing the decreased RNA level of Linc01615 after knockdown in MDAMB231 cells. Effect of Linc01615 knockdown on MDAMB231 **(B)** Cell proliferation **(C)** Colony formation ability. **(D)** Semi-quantitative and Q-PCR data showing the decreased RNA level of Linc01615 after knockdown in 143B cells. Effect of Linc01615 knockdown on MDAMB231 **(E)** Cell proliferation **(F)** Colony formation ability. The effect of Linc01615 knockdown on **(G)** MDAMB231 and **(H)** 143B cells cell migration ability. **(I)** Linc01615 was knocked down in MDAMB231 and 143B cells, and the level of mentioned protein was checked using western blot. **(J)** 143B control and Linc01615 Sh1 and sh2 cells were treated with PI3K inhibitor, Etoposide and Doxorubicin in triplicate and MTT assay was performed after 48 h.

To understand the role of Linc01615 in cancer cells, we identified the MDAMB231 and 143B cells with high expression of Linc01615 (Figure 4E). We used two shRNA constructs to knock down the Linc01615 expression in MDAMB231 and 143B cells. The knockdown of Linc01615 expression decreased the proliferation and colony formation efficiency in both the cell lines (Figures 5A–F). We also showed that the knockdown of Linc01615 significantly inhibited wound healing in both 143B and MDAMB231 cells (Figures 4G,H). The effect of Linc01615 on cell proliferation was further validated using the inducible shRNA method. Knockdown of Linc01615 using an inducible system also inhibited cell proliferation and migration (Supplementary Figures S6A–D). These observations suggest that Linc01615 expression is required for cancer development and progression. As SNAI1, SNAI2 and VIM play an essential role in cell migration, we check the effect of Linc1615 knockdown on SNAI1, SNAI2, and VIM. Interestingly, the Linc01615 knockdown decreased the expression of SNAI1 and SNAI2 in MDAMB231 and MCF7 (Figure 5I). We also noticed a slight but consistent decrease in VIM expression (Figure 5I). Surprisingly, we also found that knockdown of Linc01615 consistently decreases total AKT and TGFβ levels (Figure 5J). The decrease in TGFβ level suggests a feedback mechanism of Linc01615 and TGFβ expression.

### RP4-568C11.4 is downregulated in mesenchymal tumors and inhibits the cell proliferation

**FIGURE 6:**
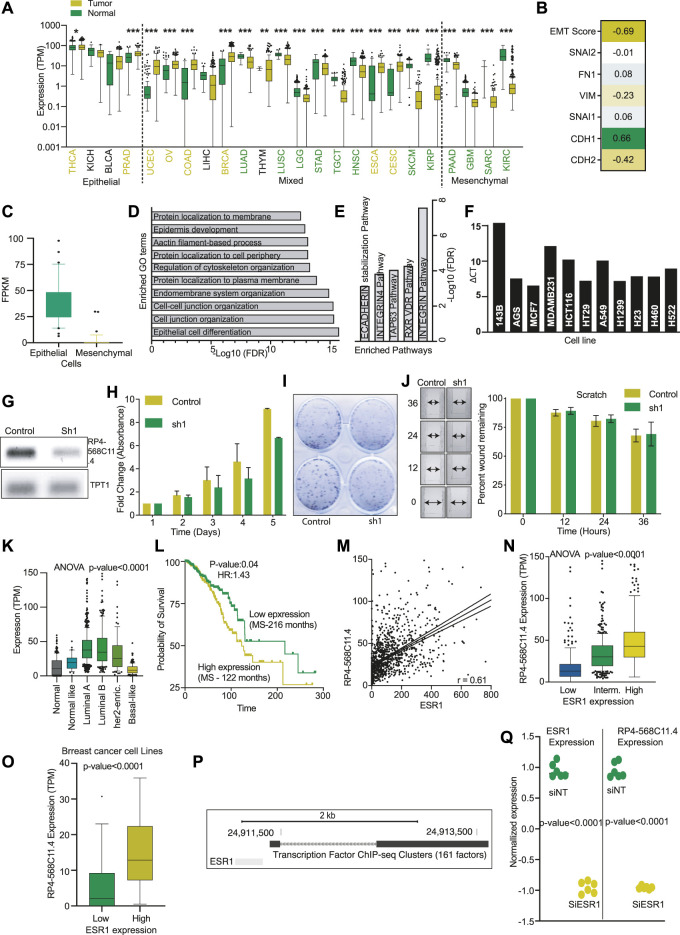
RP4-568C11.4 is an epithelial cancer gene and regulates cell proliferation. **(A)** Expression of RP4-568C11.4 was compared in all the TCGA cancer types corresponding normal and plotted. Yellow: Overexpressed in cancer, Green: Unexpressed in cancer, Black: No significant difference. **(B)** Correlation between RP4-568C11.4 and EMT markers was performed, and coefficients are represented as a heatmap. **(C)** Expression of RP4-658C11.4 in epithelial and mesenchymal cells. **(D)** Enriched GO terms and **(E)** Pathways identified using gene correlated with RP4-568C11.4 expression. **(F)** Expression of RP4-568C11.4 as identified by semi-quantitative PCR. **(G)** Expression of RP4-568C11.4 after knockdown in MCF7 cells. **(H)** Effect of RP4-568C11.4 knockdown on **(H)** Proliferation **(I)** Colony formation ability of MCF7 cells. **(J)** Effect of RP4-568C11.4 knockdown on MCF7 cell migration. **(K)** RP4-568C11.4 expression level in different subtypes of breast cancer. **(L)** Kaplan-Meier plot showing association of RP4-568C11.4 expression with breast cancer survival. **(M)** Correlation between ESR1 and RP4-568C11.4 in breast cancer. **(N)** Expression of RP4-568C11.4 in breast cancer patients with low, intermediate, or high levels of ESR1. **(O)** Expression of RP4-568C11.4 in breast cancer cells divided into low and high ESR1 levels. **(P)** UCSC genome browser view showing ESR1 binding at RP4-568C11.4 gene. **(Q)** Expression of RP4-568C11.4 and ESR1 was measured in MCF7 cells after ESR1 knockdown to show the transcription regulation of RP4-568C11.4.

Our analysis also identified another LncRNA, RP4-568C11.4, associated with epithelial tumors and under-expressed in mesenchymal cancers (Figure 6A). Expression of RP4-56811.4 was significantly positively correlated with CDH1 but showed a negative correlation with CDH2 and VIM (Figure 6B). Also, RP4-568C11.4 was down in all the high mesenchymal cells lines compared to epithelial cells (Figure 6C). We performed correlation analysis to identify the genes associated with RP4-568C11.4 expression. The identified genes were then used for the GO analysis. The most significantly enriched GO term was related to epithelial cell differentiation (Figure 6D). Also, the pathway analysis associated the RP4-568C11.4 with Integrin and E-Cadherin stabilization (Figure 6E). These results suggest that RP4-568C11.4 may affect differentiation and negatively regulate cell growth. Next, We performed PCR and identified MCF7 as cell lines with the highest expression of RP4-568C11.4 (Figure 6F). The knockdown of RP4-568C11.4 showed decreased proliferation of MCF7 cells (Figures 6G–I). However, we did not notice any significant difference in the migration of cells (Figure 6J). The expression analysis showed that RP4-568C11.4 was most significantly overexpressed in Breast cancer patients (Figure 6A). Hence, we further investigated the expression of RP4-568C11.4 in different subtypes of breast cancer. The expression of RP4-568C11.4 was highest in Luminal-A tumors and significantly less in Basal type of breast cancers (Figure 6K). Surprisingly, however, RP4-568C11.4 expression was significantly associated with poor survival of breast cancer patients (Figure 6L). As Luminal-A cancers are highly dependent on ESR1 activity, we correlated the expression of ESR1 with RP4-568C11.4, and found a strong positive correlation (Figurse 6M,N). Additionally, cell lines with higher expression of ESR1 showed high expression of RP4-568C11.4 (Figure 6O). We also found the ESR1 binding site at RP4-568C11.4 gene (Figure 6P). We compare the expression of RP4-568C11.4 in MCF7 cells treated with control or ESR1 siRNA using microarray data. The analysis showed that ESR1 knockdown cells have significantly lower expression of RP4-568C11.4 expression (Figure 6Q). These observations suggests that ESR1 regulates RP4-568C11.4 expression at least in breast cancer and RP4-568C11.4 regulates cell proliferation but not cell migration.

## Discussion

EMT plays a crucial role in cancer progression. The part of EMT markers like VIM, FN1, SNAI1, SNAI2, CDH1, CDH2, etc., are well documented (Zeisberg and Neilson, 2009). There have been studies where expression of all the mesenchymal genes and epithelial genes were used to calculate the EMT score, and patients were classified as high or low EMT (Gibbons and Creighton, 2018). However, the Expression pattern of these genes was not sufficient to classify the cancer types into EMT classes. Here, we identified 14 LncRNAs strongly correlating EMT markers to calculate the EMT score. The survival correlation of mesenchymal and epithelial LncRNAs suggests that mesenchymal cells are more aggressive than epithelial cells (Figure 1E). Similarly, significant poor survival of patients with high EMT scores showed that patients with high mesenchymal nature could be more aggressive. The aggressiveness of tumors with more EMT scores could be due to high metastatic potential or higher resistance to the therapy. This outcome is supported by earlier reports suggesting that patients with high EMT show increased resistance to chemotherapy (Fischer et al., 2015; Zheng et al., 2015). Further, we classify the cancer types into various EMT groups. We found a majority of cancers have a range of EMT scores; or in other words, cancers have variations in EMT nature. However, some cancers are strongly epithelial (THCA, KICH, BLCA, PRAD) or mesenchymal (CHOL, PAAD, GBM, MESO, DLBC, PCPG, SARC, and KIRC) in nature. Interestingly, there was no significant correlation between the EMT score of the tumors with the tissue of origin. For example, while KIRC was highly mesenchymal compared to BLCA, normal Kidney and normal bladder samples did not show a significant difference in EMT score. Interestingly, while VIM expression was different in cancer types depending on EMT nature, CDH1 expression did not correlate with the EMT nature of the cancers. Suggesting VIM is a better reporter of the EMT than CDH1. Similar to cancer classification, cell lines were also divided into mesenchymal and epithelial groups. We have reported cells with high mesenchymal and epithelial nature, which can prove significant in selecting cell lines for the EMT assays. We also showed that Mesenchymal cells were more resistant to the general chemotherapeutic agents than epithelial cells, validating the earlier findings. Earlier reports have suggested that mesenchymal cells are dependent on the PI3K-AKT pathway to maintain proliferation in the absence of serum (Xu et al., 2015). Authors have also shown that mesenchymal cells have high PI3K-AKT activity *in vivo*. In our analysis, we also found that mesenchymal cells are highly dependent upon the PI3K-AKT signalling. The analysis with drug sensitivity data also showed that PI3K signalling pathway inhibitors have a higher sensitivity to the PI3K pathway inhibitor than Epithelial cells, which showed increased sensitivity to general chemotherapeutic drugs (drugs inducing DNA damages, ROS, etc.).

The expression comparison of genes between epithelial and mesenchymal cells identified a novel LncRNA, Linc01615, as mesenchymal specific LncRNA. Linc01615 was positively correlated with EMT score and showed a high expression majority of mesenchymal cancers, and was associated with poor prognosis, suggesting the role of Linc01615 on the mesenchymal phenotype. The knockdown of Linc01615 also inhibited the total TGFβ and AKT. Our experiments also showed TGFβ mediated regulation of the Linc01615 expression. These findings indicate the feedback loop between TGFβ and Linc01615.

RP4-568C11.4 was another LncRNA that was identified as epithelial cancer-associated LncRNAs. Expression of RP4-658C11.4 was high in Luminal A breast cancers compared to normal and Basal cancers. Also, knockdown of RP4-568C11.4 inhibited only cell proliferation with no significant effect on migration. These results indicate that RP4-568C11.4 may be required for the development of tumors but not required for the progression of tumors.

## Conclusion

In summary, we have developed an EMT signature and classified the tumors based on the EMTness of each cancer. We have shown that tumors with high mesenchymal nature tend to survive poorly. The PI3K pathway plays a crucial role in determining the Mesenchymal nature of the tumors. We also found that mesenchymal cells are more sensitive to PI3K inhibition than epithelial cells. Lastly, we have identified two novel cancer-linked LncRNAs with mesenchymal and epithelial cancer associations.

## Data Availability

The datasets presented in this study can be found in online repositories. The names of the repository/repositories and accession number(s) can be found in the article/Supplementary Material.

## Abbreviations

EMT, Epithelial to mesenchymal transitions; LncRNA, Long non-coding RNA; TCGA, The Cancer Genome atlas.
