# Supplementary material for: An epithelial-mesenchymal plasticity signature identifies two novel LncRNAs with the opposite regulation
Source: Front Cell Dev Biol. 2022 Sep 1;10:885785. doi: 10.3389/fcell.2022.885785 (PMC9474898; doi:10.3389/fcell.2022.885785)
Supplement: Supplementary file 3 [file DataSheet1.PDF]

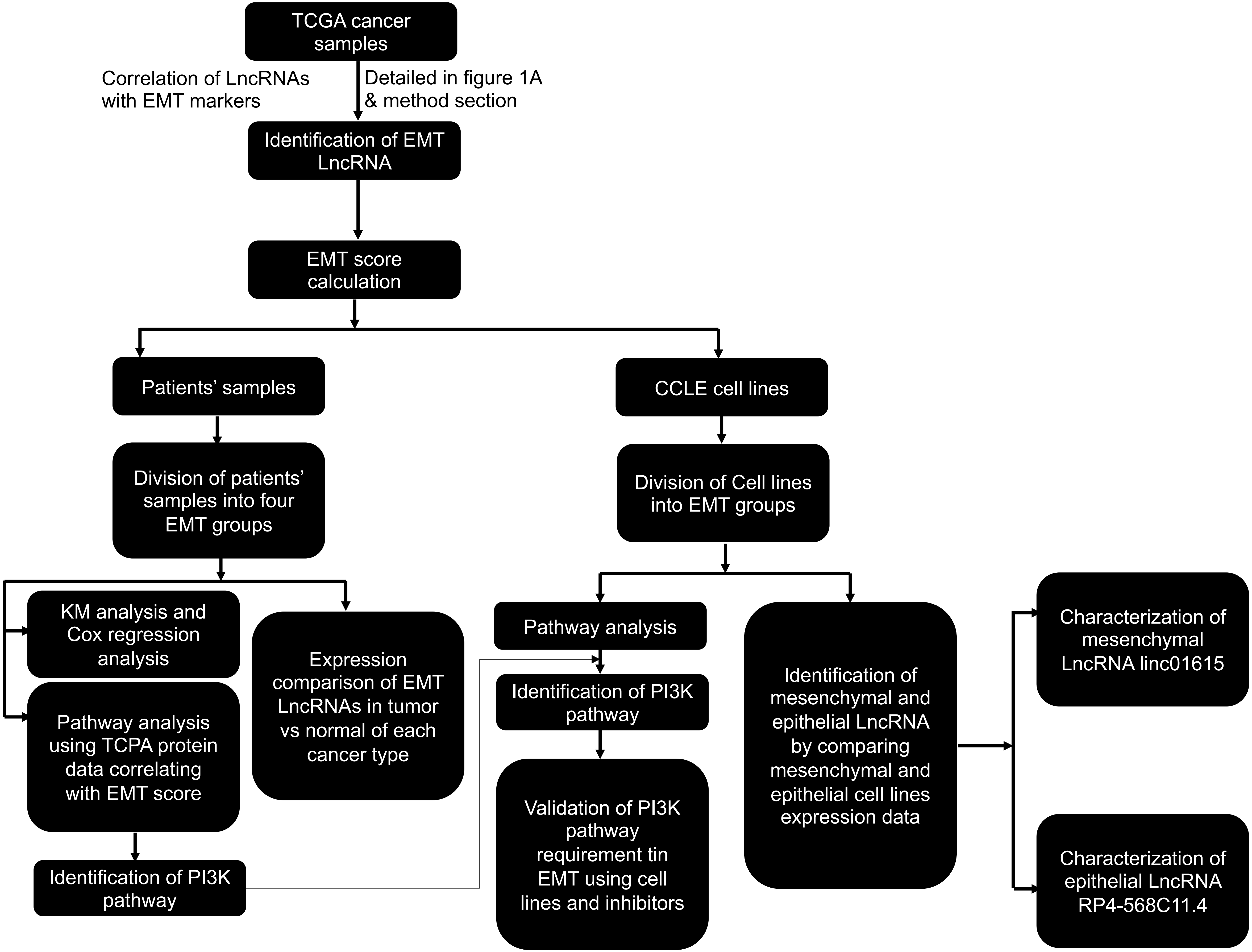
TCGA cancer samples
Correlation of LncRNAs with EMT markers
Detailed in figure 1A & method section
Identification of EMT LncRNA
EMT score calculation
Patients' samples
CCLE cell lines
Division of patients' samples into four EMT groups
Division of Cell lines into EMT groups
KM analysis and Cox regression analysis
Pathway analysis using TCPA protein data correlating with EMT score
Expression comparison of EMT LncRNAs in tumor vs normal of each cancer type
Identification of PI3K pathway
Pathway analysis
Identification of PI3K pathway
Validation of PI3K pathway requirement tin EMT using cell lines and inhibitors
Identification of mesenchymal and epithelial LncRNA by comparing mesenchymal and epithelial cell lines expression data
Characterization of mesenchymal LncRNA linc01615
Characterization of epithelial LncRNA RP4-568C11.4

Supplementary figure 2

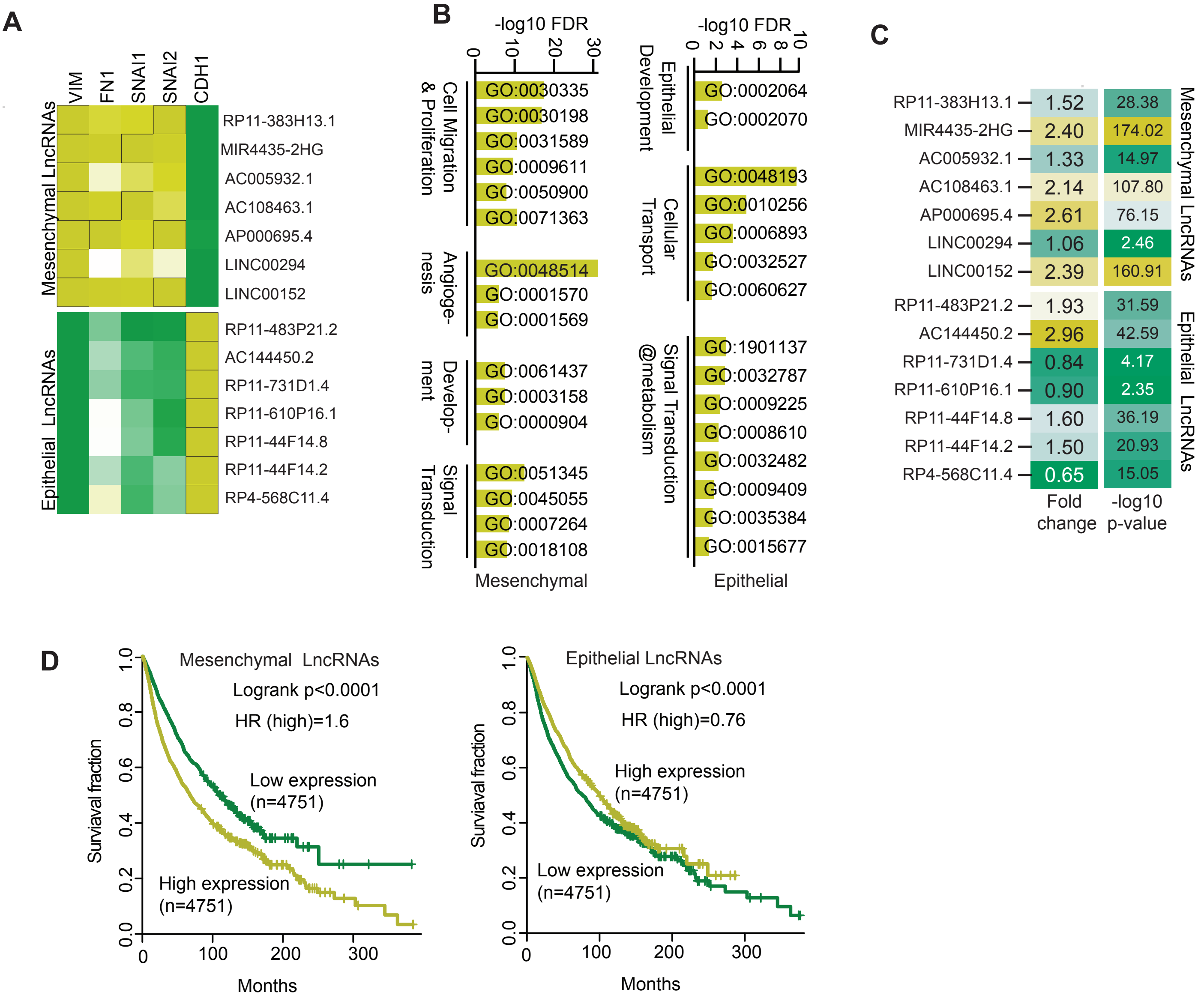

Supplementary Figure 2: **A)** The EMT associated LncRNAs were divided into epithelia and mesenchymal groups and correlation coefficient with given markers were calculated and plotted as heatmap. Green shows low correlation, yellow shows high correlation. **B)** Mesenchymal and epithelial LncRNAs expression were correlated with all the proteins coding genes in 32 cancer types and Gene ontology analysis was performed and significant GO terms were plotted. **C**) The expression of all the 14 LncRNAs was compared in pooled normal and pooled cancers (32 types) and fold change and p-values were plotted. **D**) The 32 cancer patients were divided into low and high expression of combined mesenchymal or epithelial LncRNAs (using Gepia2 databases) and survival analysis was performed. The KM plots are showing the difference in survival of high and low expressing groups of mesenchymal and epithelial LncRNAs.

Supplementary Figure 3

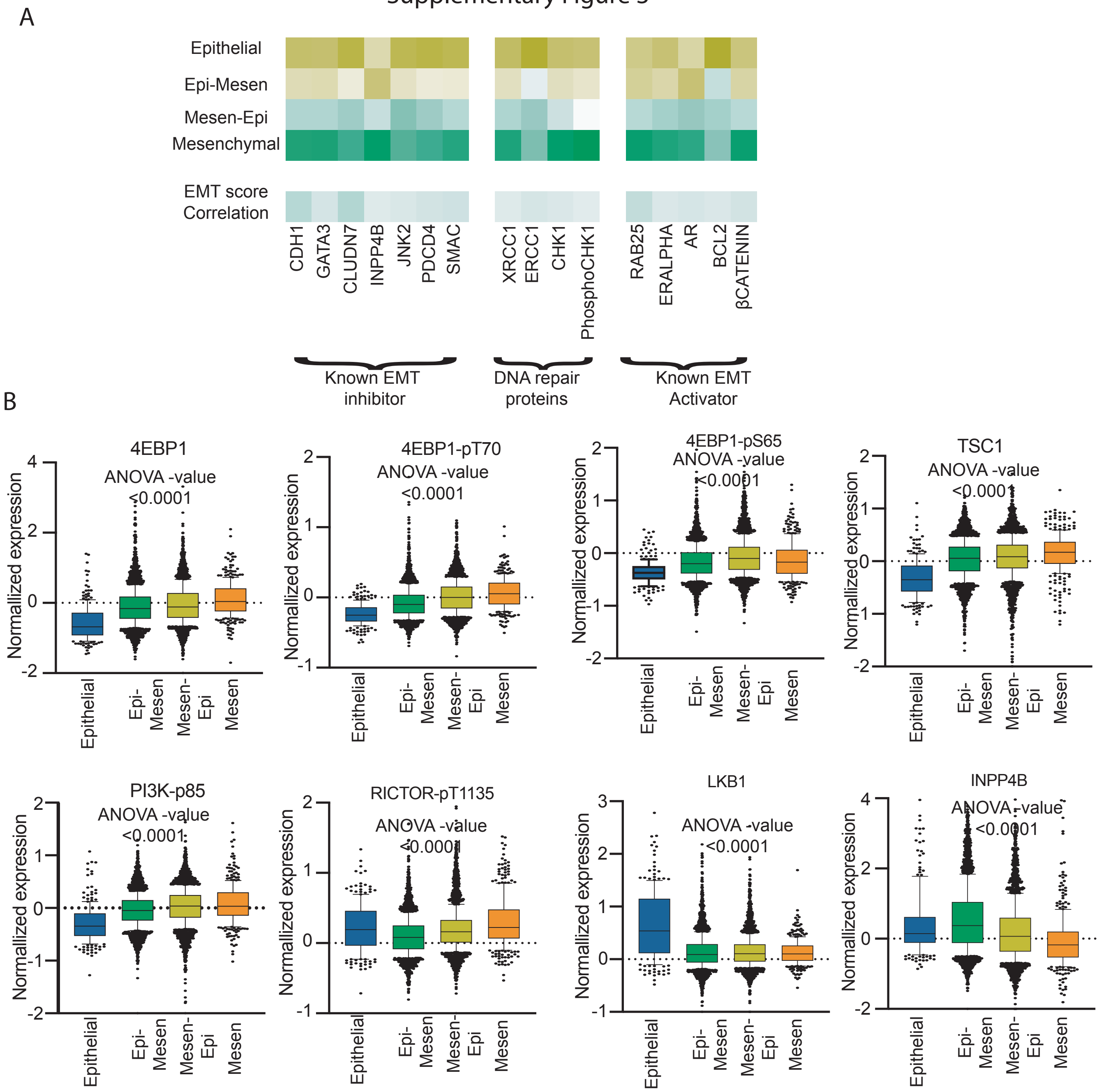

Supplementary figure 3: A) The average expression of proteins with negative correlation with EMT score was plotted in heatmap in for EMT classes. The proteins are divided into three classes depending upon their role in EMT and DNA repair. **B)** Box plots showing the expression of various PI3K pathway proteins in for EMT classes.

Supplementary Figure 4

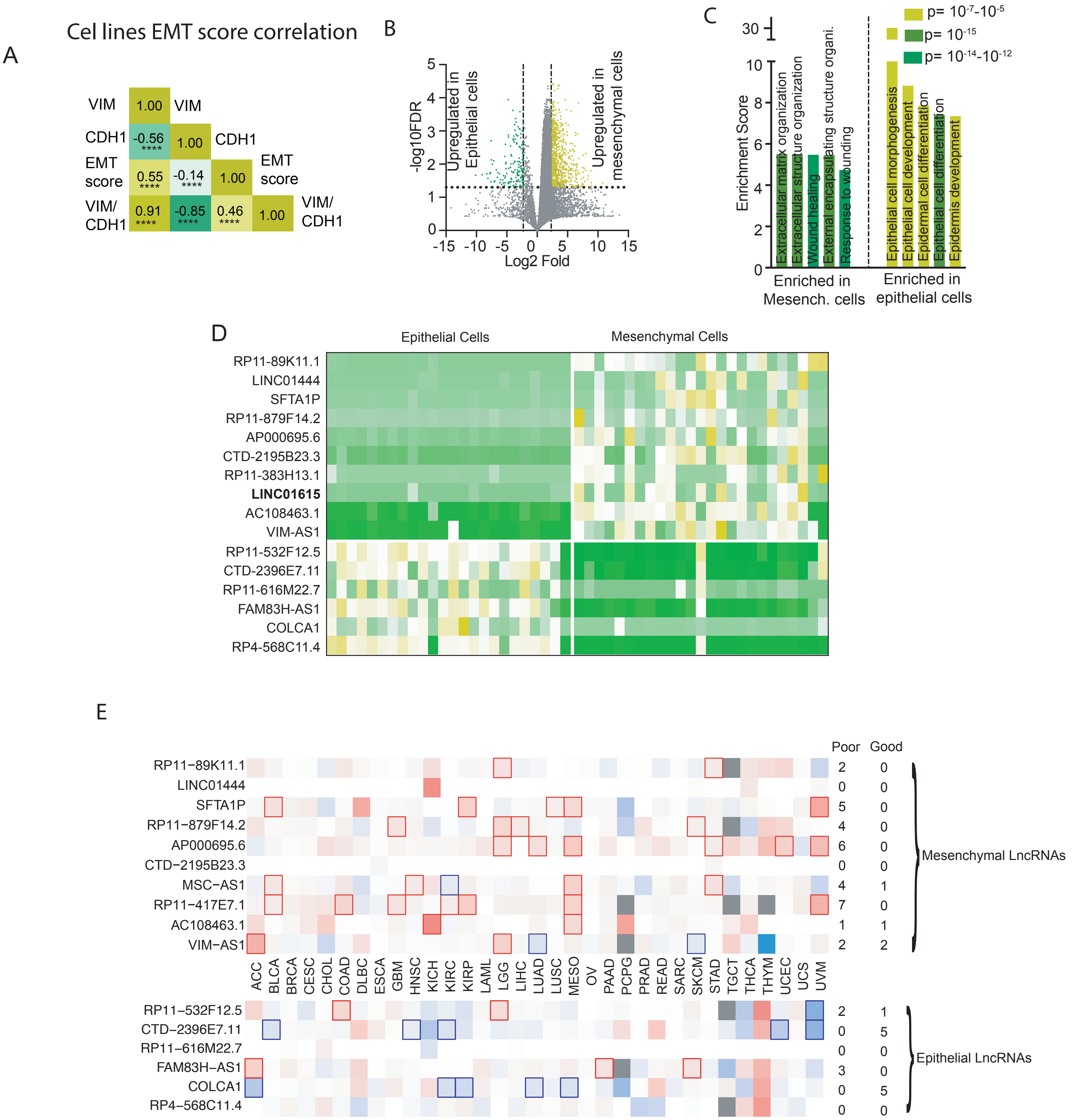

Supplementary Figure 4: A). The Heatmap showing the correlation of EMT score with VIM and CDH1 in CCLE cell lines. **B**) The expression of genes was compared between mesenchymal and Epithelial cell lines and results were plotted as volcano plot. **C**) The result of pathway analysis to show the enriched pathways in mesenchymal and epithelial cells. **D**) The expression of top LncRNAs with high expression in Epithelial cells or mesenchymal cells are plotted as heatmap. **E)** Heatmap representing the prognostic role of LncRNAs specific to mesenchymal cell lines and epithelial cell lines.

Supplementary data 5

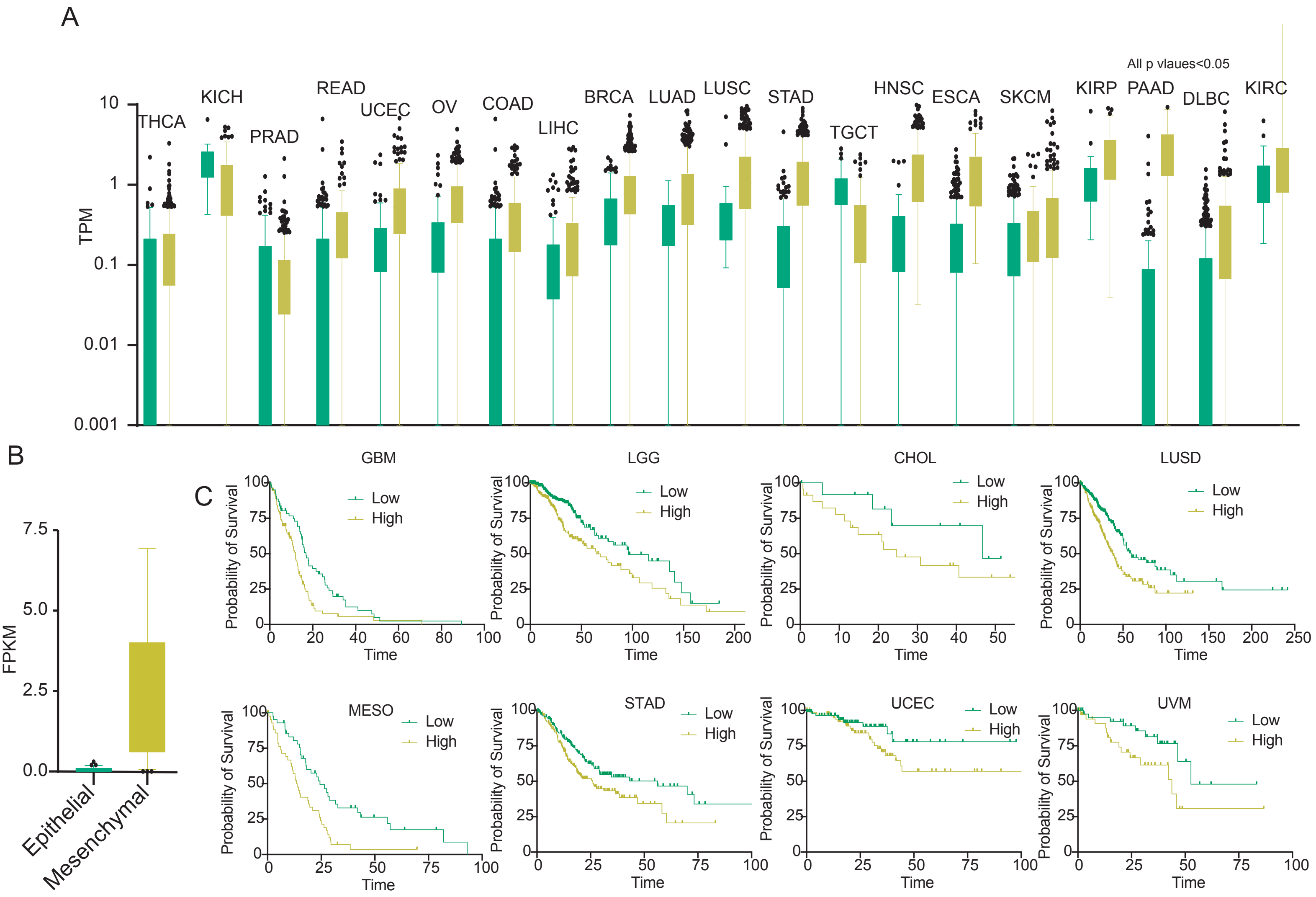

Supplementary Figure 5: A) Expression of AP000695.6 was compared in all the TCGA cancer type and corresponding normal and plotted. **B)** Expression of AP000695.6 in epithelial and mesenchymal cell lines. **C)** Individual cancer types were divided into low and high AP000695.6 groups and survival was compared using Kaplan-Meier analysis.

Supplementary data 6

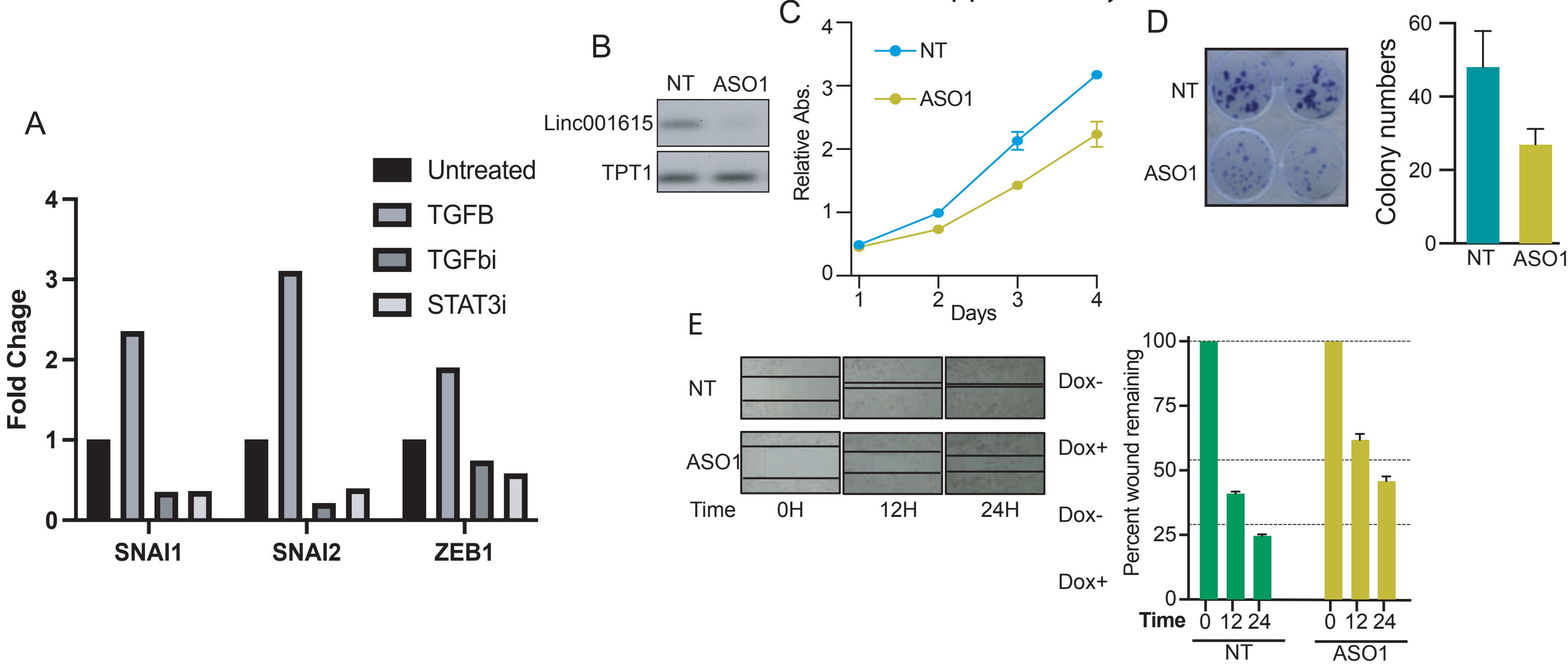

Supplementary Figure 6: ASO experiment to validate Linc01615 is required for cell proliferation and cell migration:A) Effect of given treatment on EMT markers B) Semi-quantitative and Q-PCR data showing the decreased RNA level of Linc01615 after knockdown in 143B cells using ASO. Effect of Linc01615 knockdown on 143B C) Cell proliferation D) Colony formation ability. E) 143B cells cell migration ability.
